# Supplementary material for: OTULIN inhibits RIPK1-mediated keratinocyte necroptosis to prevent skin inflammation in mice
Source: Nat Commun. 2021 Oct 8;12:5912. doi: 10.1038/s41467-021-25945-1 (PMC8501112; doi:10.1038/s41467-021-25945-1)
Supplement: Supplementary file 1 — Supplementary Information [file 41467_2021_25945_MOESM1_ESM.pdf]

SUPPLEMENTARY INFORMATION

OTULIN inhibits RIPK1-mediated keratinocyte necroptosis to prevent skin inflammation in mice

Hannah Schünke[1,2], Ulrike Goebel[2], Ivan Dikic[3], Manolis Pasparakis[1,2,4]

[1]Institute for Genetics, University of Cologne, Cologne, Germany.
[2]Cologne Excellence Cluster on Cellular Stress Responses in Aging-Associated Diseases (CECAD), University of Cologne, Cologne, Germany.
[3]Institute of Biochemistry II, Goethe-Universität Frankfurt am Main, Buchmann Institute for molecular life sciences, Frankfurt, Germany.
[4]Center for Molecular Medicine Cologne (CMMC), University of Cologne, Cologne, Germany.

Correspondence: pasparakis@uni-koeln.de

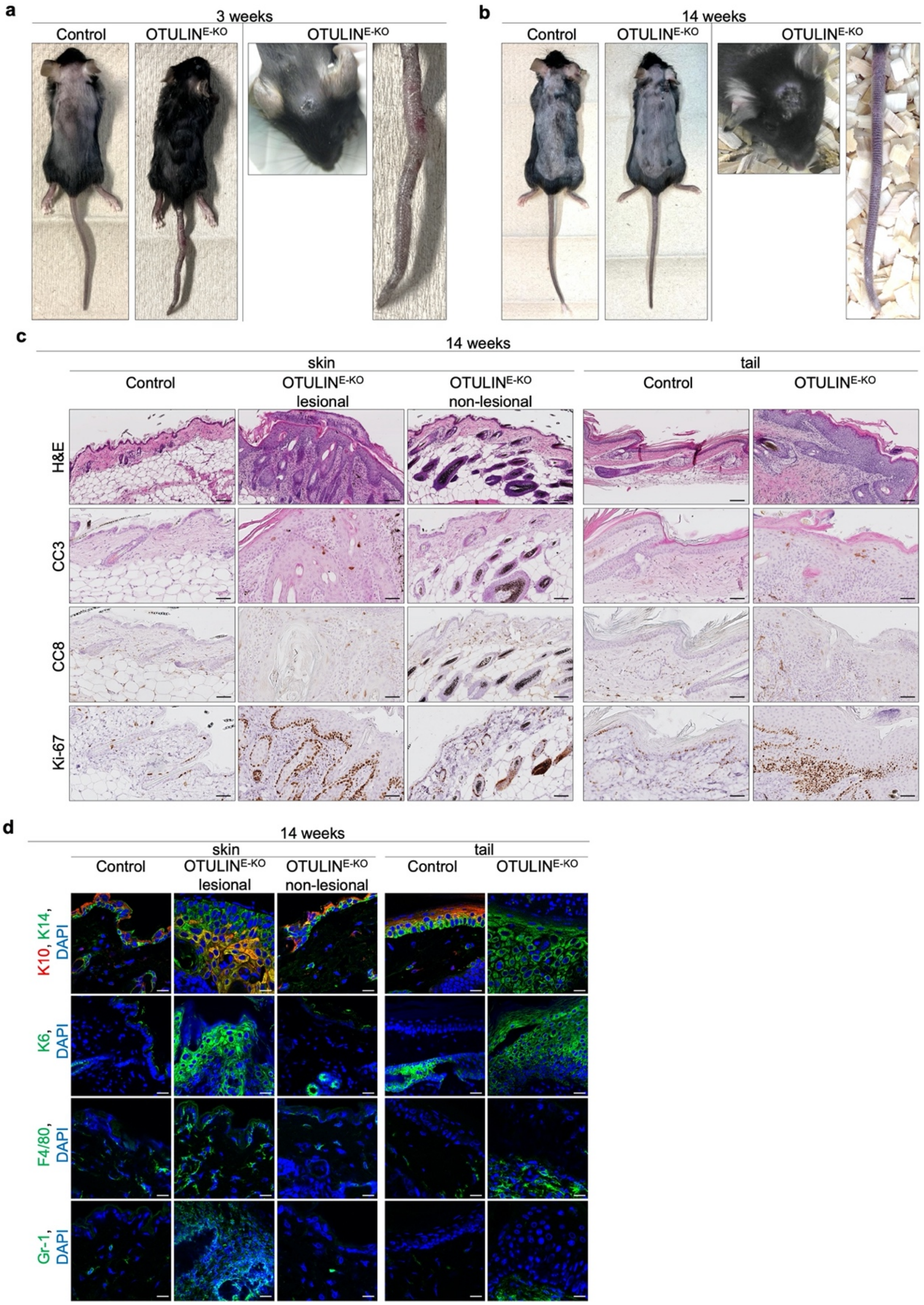

Supplementary Figure 1. Mice with keratinocyte-specific OTULIN knockout develop skin inflammation. a, b Photographs of mice with the indicated genotypes at the age of 3 weeks (**a**) and 14 weeks (**b**). Images shown are representative of n > 25 OTULIN^{E-KO} mice. **c** Representative images of skin sections from 14-week old mice of the indicated genotypes (n>5), stained with haematoxylin and eosin (H&E), or immunostained with antibodies against cleaved caspase 3 (CC3), cleaved caspase 8 (CC8) or Ki67. Scale barS: H&E = 100µm; CC3, CC8, Ki67 = 50µm. **d** Representative images of skin sections from 14-week old mice of the indicated genotypes (n>5), immunostained with antibodies against keratin 10 (K10), keratin 14 (K14), keratin 6 (K6), F4/80, Gr-1 and counterstained with DAPI (DNA stain). Scale bars: K10, K14, K6 = 20µm; F4/80, Gr-1 = 30µm.

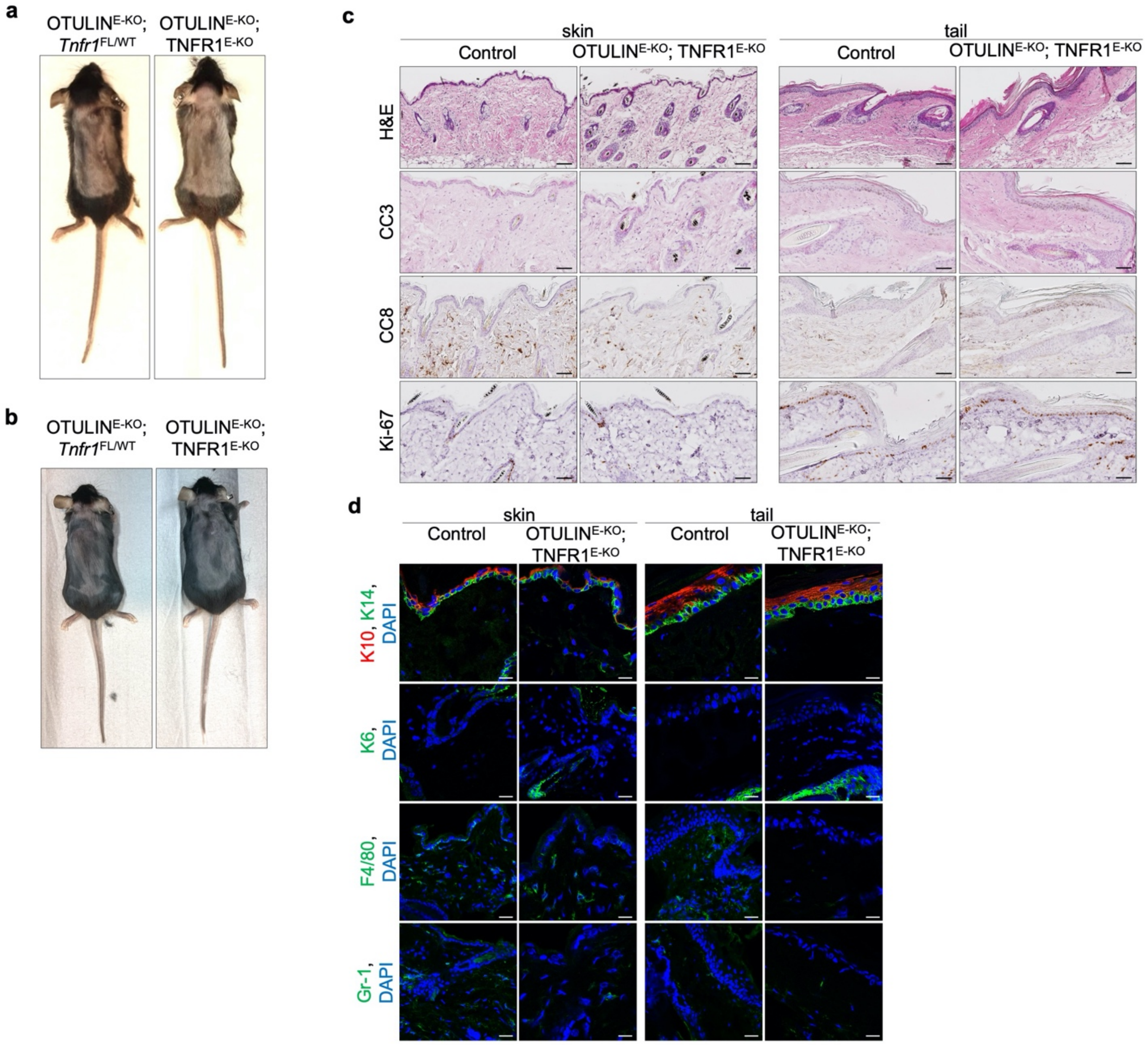

Supplementary Figure 2. Keratinocyte-specific TNFR1 deficiency prevents skin inflammation in OTULIN$^{E\text{-}KO}$ mice. a, b Photographs of mice with the indicated genotypes at the age of 3 weeks (**a**) and 50 weeks (**b**). Images shown are representative of n > 5 mice with the indicated genotypes.

c Representative images from skin sections from 50-week old mice of the indicated genotypes (n=3), stained with H&E, or immunostained with anti-CC3, anti-CC8 or anti-Ki67 antibodies. Scale bars: H&E = 100µm; CC3, CC8, Ki67 = 50µm. **d** Representative images from skin sections from 50-week old mice of the indicated genotypes (n=3), immunostained with anti-keratin 10, anti-K14, anti-K6, anti-F4/80, anti-Gr-1 antibodies and counterstained with DAPI (DNA stain). Scale bars: K10, K14, K6 = 20µm; F4/80, Gr-1 = 30µm.

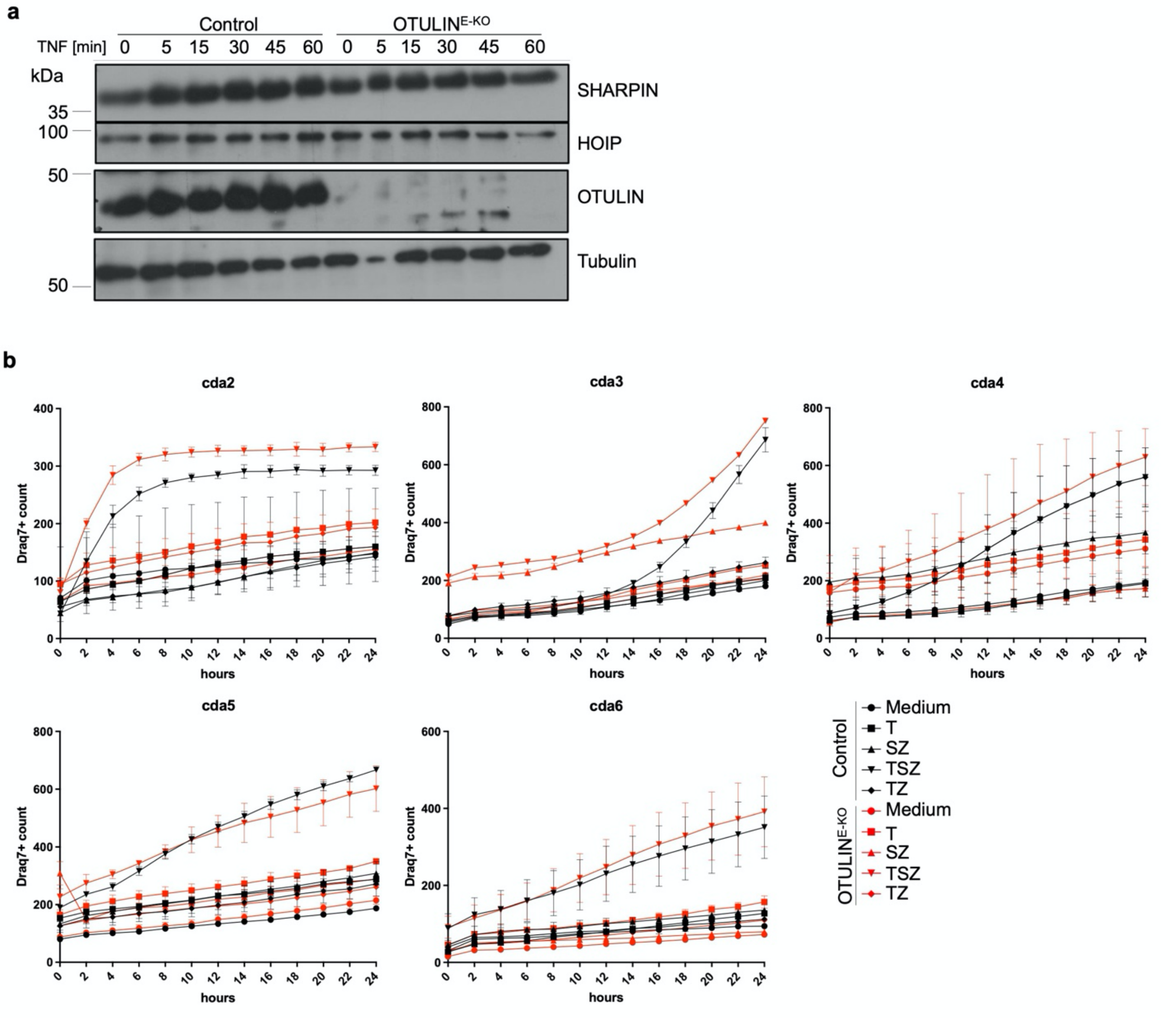

Supplementary Figure 3. TNF-induced cell death responses in OTULIN-deficient keratinocytes. a Immunoblot analysis with the indicated antibodies of protein extracts from primary keratinocytes derived from OTULIN$^{E\text{-}KO}$ mice or WT mice stimulated with TNF for the indicated timepoints. **b** Cell death measured by Draq7 uptake in primary keratinocytes from OTULIN$^{E\text{-}KO}$ and WT mice treated with combinations of TNF (T), the SMAC mimetic Birinapant (S) or z-VAD-fmk (Z) for 24 hours. Graphs show mean values from technical duplicates. Five replicate experiments shown to support Figure 3d.

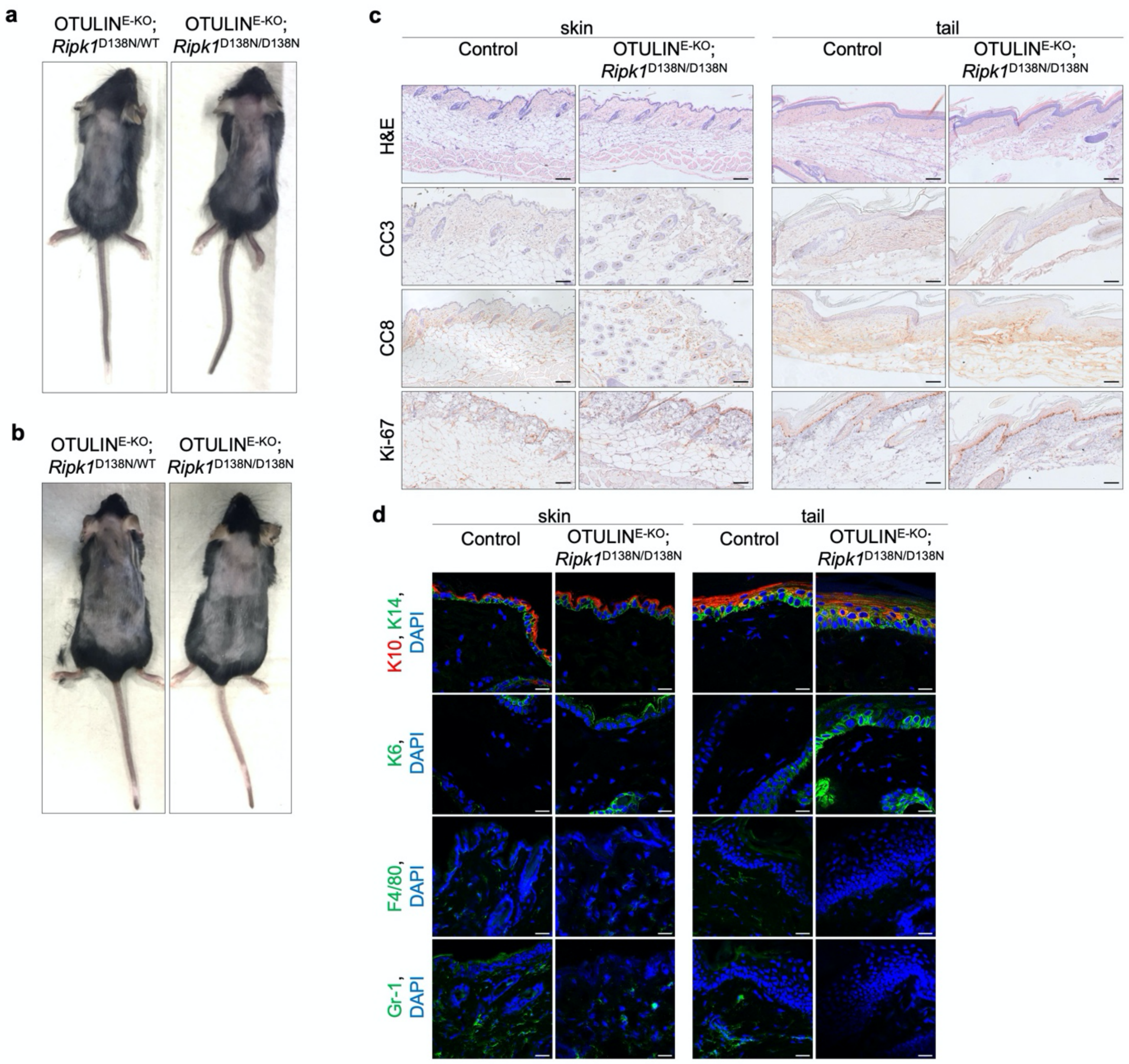

Supplementary Figure 4. Inhibition of RIPK1 kinase activity by crossing to *Ripk1*$^{\mathbf{D138N/D138N}}$ mice prevents skin inflammation in OTULIN$^{\mathbf{E\text{-}KO}}$ mice. a, b Photographs of mice with the indicated genotypes at the age of 3 weeks (**a**) and 50 weeks (**b**). Images shown are representative of n > 5 mice with the indicated genotypes. **c** Representative images from skin sections from 50-week old mice of the indicated genotypes (n=3), stained with H&E, or immunostained with anti-CC3, anti-CC8 or anti-Ki67 antibodies. Scale bars: H&E = 100µm; CC3, CC8, Ki67 = 50µm. **d** Representative images from skin sections from 50-week old mice of the indicated genotypes (n=3), immunostained with anti-keratin 10, anti-K14, anti-K6, anti-F4/80, anti-Gr-1 antibodies and counterstained with DAPI (DNA stain). Scale bars: K10, K14, K6 = 20µm; F4/80, Gr-1 = 30µm.

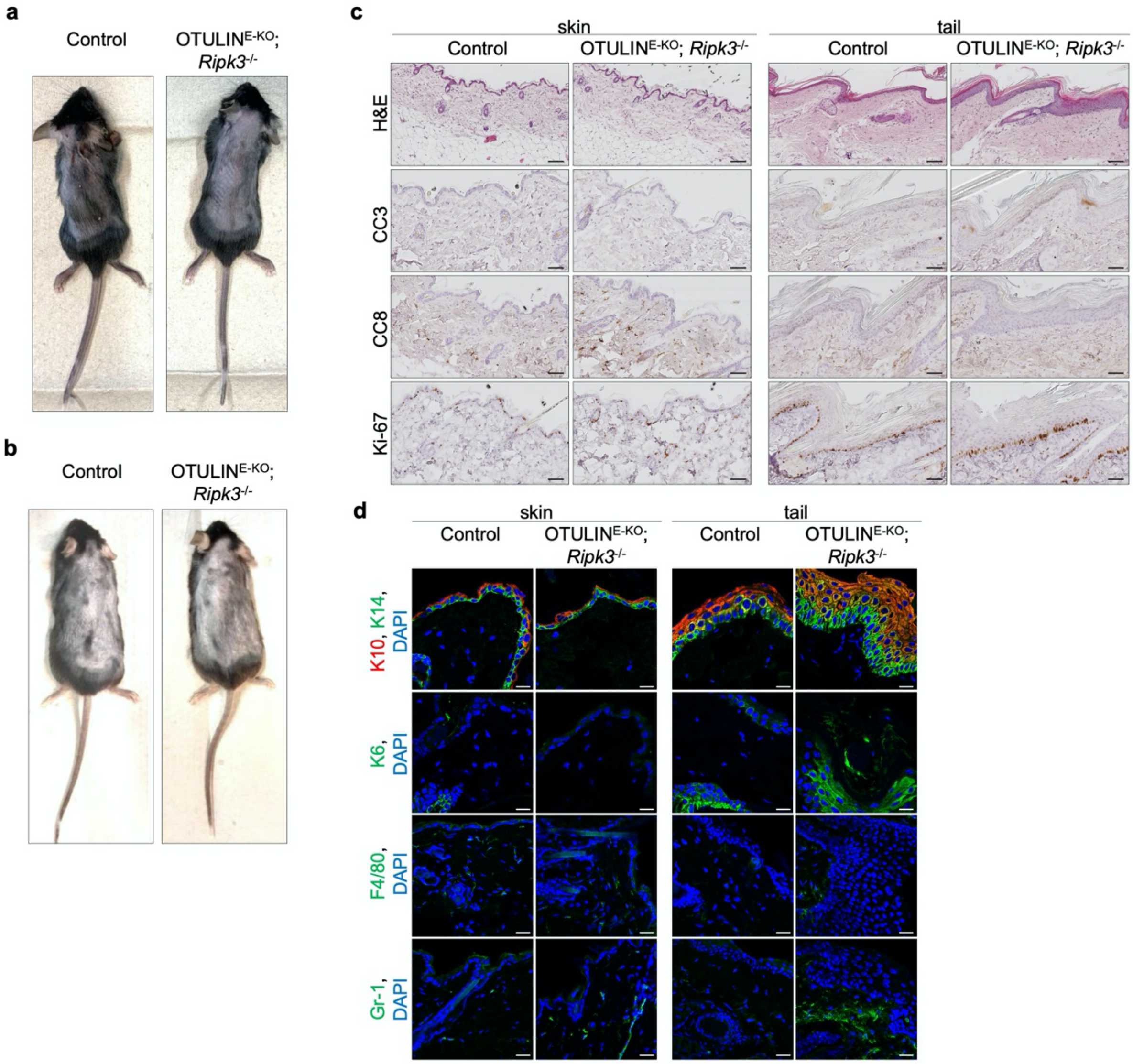

Supplementary Figure 5. RIPK3 deficiency strongly protects OTULIN$^{E\text{-}KO}$ mice from skin lesion development. a, b Photographs of mice with the indicated genotypes at the age of 3 weeks (**a**) and 50 weeks (**b**). Images shown are representative of n > 5 mice with the indicated genotypes. **c** Representative images from skin sections from 50-week old mice of the indicated genotypes (n=3), stained with H&E, or immunostained with anti-CC3, anti-CC8 or anti-Ki67 antibodies. Scale bars: H&E = 100µm; CC3, CC8, Ki67 = 50µm. **d** Representative images from skin sections from 50-week old mice of the indicated genotypes (n=3), immunostained with anti-keratin 10, anti-K14, anti-K6, anti-F4/80, anti-Gr-1 antibodies and counterstained with DAPI (DNA stain). Scale bars: K10, K14, K6 = 20µm; F4/80, Gr-1 = 30µm.

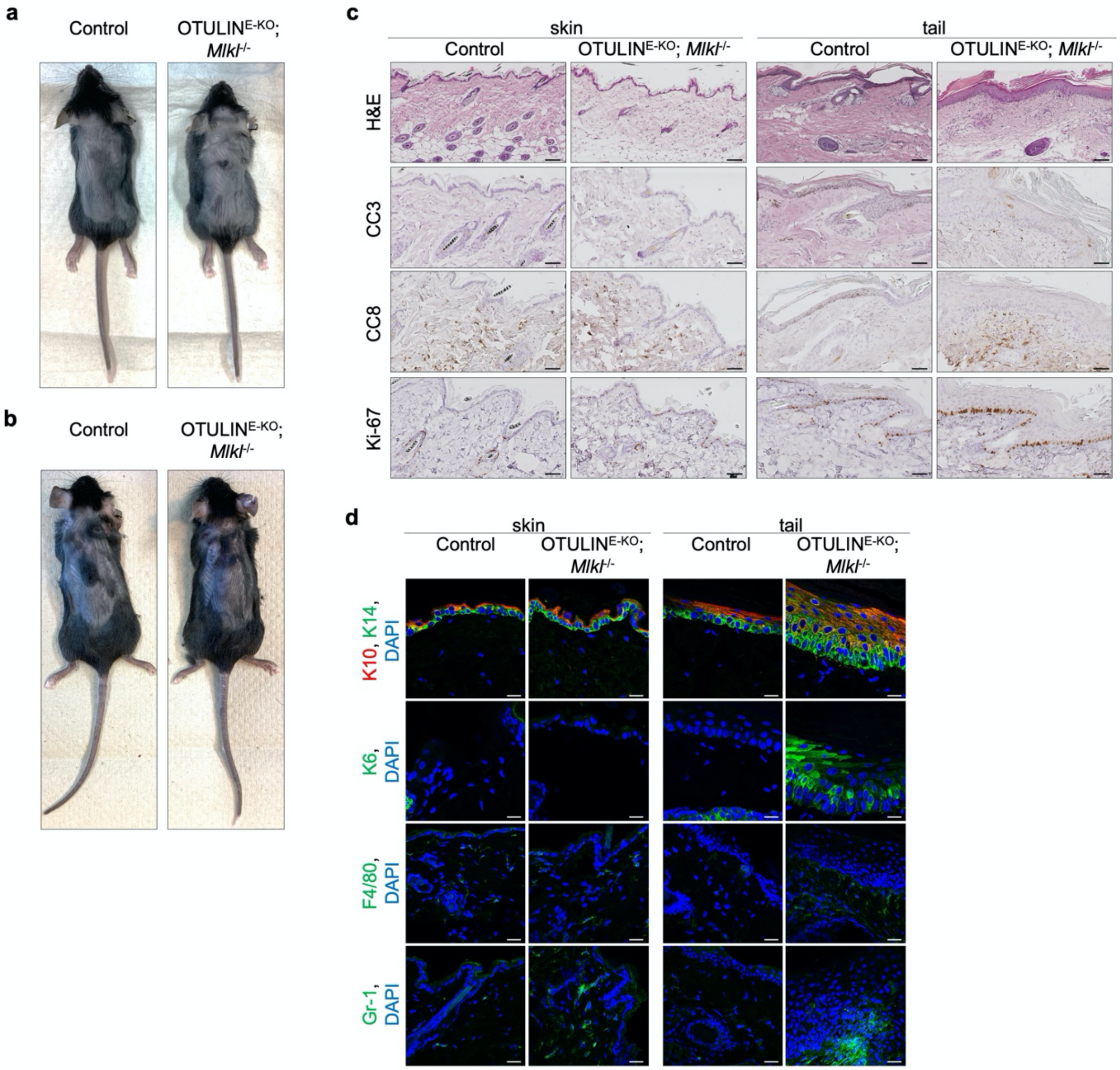

Supplementary Figure 6. MLKL deficiency strongly delays and ameliorates skin lesion development in OTULIN$^{E\text{-}KO}$ mice. a, b Photographs of mice with the indicated genotypes at the age of 3 weeks (**a**) and 50 weeks (**b**). Images shown are representative of n > 5 mice with the indicated genotypes.

c Representative images from skin sections from 50-week old mice of the indicated genotypes (n=3), stained with H&E, or immunostained with anti-CC3, anti-CC8 or anti-Ki67 antibodies. Scale bars: H&E = 100µm; CC3, CC8, Ki67 = 50µm. **d** Representative images from skin sections from 50-week old mice of the indicated genotypes (n=3), immunostained with anti-keratin 10, anti-K14, anti-K6, anti-F4/80, anti-Gr-1 antibodies and counterstained with DAPI (DNA stain). Scale bars: K10, K14, K6 = 20µm; F4/80, Gr-1 = 30µm.

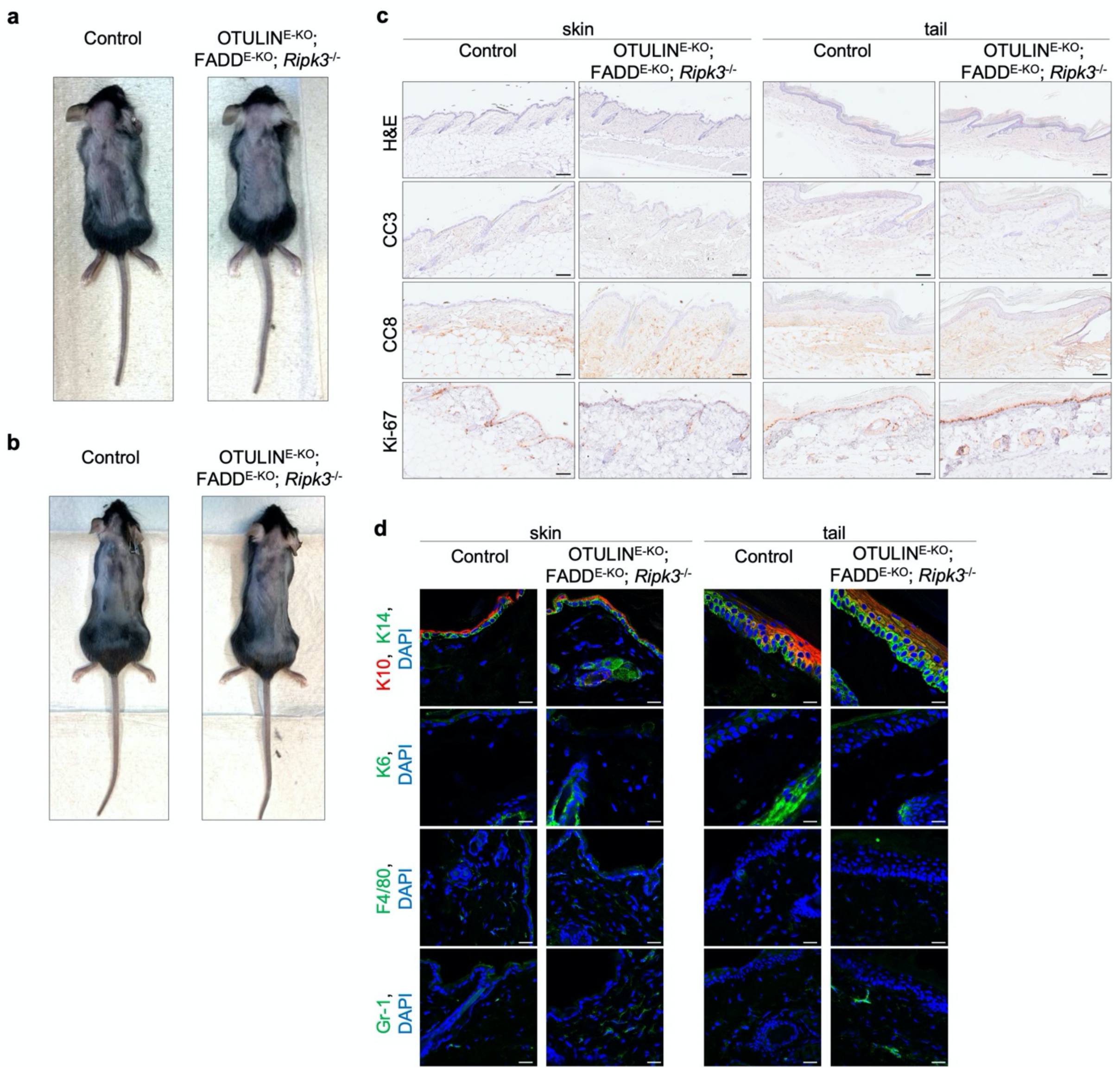

Supplementary Figure 7. Combined inhibition of necroptosis and apoptosis fully prevents skin inflammation in OTULIN$^{E\text{-}KO}$ mice. a, b Photographs of mice with the indicated genotypes at the age of 3 weeks (**a**) and 50 weeks (**b**). Images shown are representative of n > 5 mice with the indicated genotypes. **c** Representative images from skin sections from 50-week old mice of the indicated genotypes (n=3), stained with H&E, or immunostained with anti-CC3, anti-CC8 or anti-Ki67 antibodies. Scale bars: H&E = 100µm; CC3, CC8, Ki67 = 50µm. **d** Representative images from skin sections from 50-week old mice of the indicated genotypes (n=3), immunostained with anti-keratin 10, anti-K14, anti-K6, anti-F4/80, anti-Gr-1 antibodies and counterstained with DAPI (DNA stain). Scale bars: K10, K14, K6 = 20µm; F4/80, Gr-1 = 30µm.

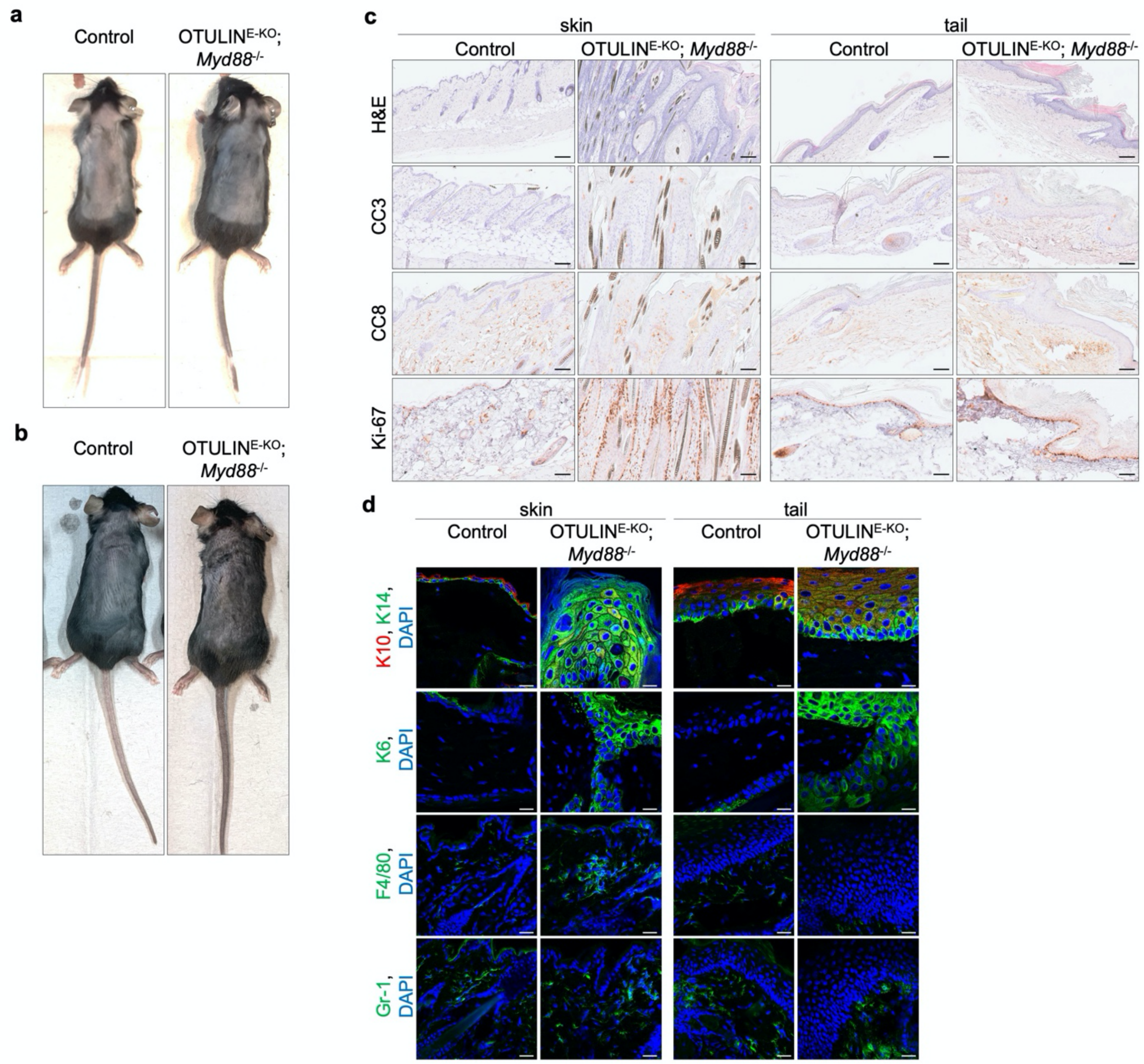

Supplementary Figure 8. MyD88 deficiency delays and ameliorates the development of skin inflammation in OTULIN^{E-KO} mice. a, b Photographs of mice with the indicated genotypes at the age of 3 weeks (**a**) and 50 weeks (**b**). Images shown are representative of n > 5 mice with the indicated genotypes. **c** Representative images from skin sections from 50-week old mice of the indicated genotypes (n=3), stained with H&E, or immunostained with anti-CC3, anti-CC8 or anti-Ki67 antibodies. Scale bars: H&E = 100µm; CC3, CC8, Ki67 = 50µm. **d** Representative images from skin sections from 50-week old mice of the indicated genotypes (n=3), immunostained with anti-keratin 10, anti-K14, anti-K6, anti-F4/80, anti-Gr-1 antibodies and counterstained with DAPI (DNA stain). Scale bars: K10, K14, K6 = 20µm; F4/80, Gr-1 = 30µm.

Biological process	GO ID	P-value	P-value (adj.)	Gene ID
Defense response to virus	GO:0051607	2.0936E-10	5.1566E-07	Ifit1/Oasl2/Rtp4/Ifitm3/Ddx60/Stat1/Ifih1/Isg15/Oas1c/Oas1b/Trim34a/Spn/Irf7/Stat2/Bst2/Rsad2/Dtx3l/Eif2ak2
Response to virus	GO:0009615	5.512E-10	6.7881E-07	Ifit1/Oasl2/Rtp4/Ifitm3/Ifi27l2a/Ddx60/Stat1/Ifih1/Isg15/Oas1c/Oas1b/Trim34a/Spn/Irf7/Stat2/Bst2/Rsad2/Dtx3l/Eif2ak2
Negative regulation of viral genome replication	GO:0045071	4.2068E-07	0.00034538	Oasl2/Ifitm3/Isg15/Oas1c/Oas1b/Bst2/Rsad2/Eif2ak2
Negative regulation of viral process	GO:0048525	3.3444E-06	0.00205932	Oasl2/Ifitm3/Stat1/Isg15/Oas1c/Oas1b/Bst2/Rsad2/Eif2ak2
Negative regulation of viral life cycle	GO:1903901	6.4135E-06	0.00298786	Oasl2/Ifitm3/Isg15/Oas1c/Oas1b/Bst2/Rsad2/Eif2ak2
Innate immune response	GO:0045087	7.2786E-06	0.00298786	Ifit1/Oasl2/Ifitm3/Sdhaf4/Stat1/Atg9a/Ifih1/Gbp2/Isg15/Gbp9/Herc6/Irf7/H60c/Stat2/Bst2/Pstpip1/Rsad2/Parp14/Dtx3l/Eif2ak2/Usp14
Regulation of viral life cycle	GO:1903900	1.2064E-05	0.00424492	Oasl2/Ifitm3/Pcx/Isg15/Oas1c/Oas1b/Hs3st5/Bst2/Rsad2/Eif2ak2
Regulation of viral genome replication	GO:0045069	1.7951E-05	0.00538435	Oasl2/Ifitm3/Isg15/Oas1c/Oas1b/Bst2/Rsad2/Eif2ak2
Regulation of viral process	GO:0050792	1.9675E-05	0.00538435	Oasl2/Ifitm3/Pcx/Stat1/Isg15/Oas1c/Oas1b/Hs3st5/Bst2/Rsad2/Eif2ak2
Response to bacterium	GO:0009617	2.5676E-05	0.00632402	Ifit1/Ifi44/Usp18/Stat1/B2m/Adh5/Gbp2/Isg15/Gbp9/Herc6/Spn/2610528A11Rik/Rnase1/Hba-a1/Pmp22/H2-K1/Psmb9/Eif2ak2/Cd274
Response to interferon-beta	GO:0035456	3.5515E-05	0.0071472	Ifit1/Ifitm3/Stat1/Ifi207/Gbp2/Bst2
Regulation of interspecies interactions between organisms	GO:0043903	3.6901E-05	0.0071472	Oasl2/Ifitm3/Pcx/Stat1/Isg15/Oas1c/Oas1b/Hs3st5/Bst2/Rsad2/Eif2ak2
Regulation of T cell mediated immunity	GO:0002709	3.9879E-05	0.0071472	Traf2/B2m/Spn/Rsad2/H2-K1/H2-D1/H2-Q6
Sterol biosynthetic process	GO:0016126	4.0626E-05	0.0071472	Hsd17b7/Lbr/Cyp51/Msmo1/Sec14l2/Hmgcr
Positive regulation of T cell mediated immunity	GO:0002711	4.6309E-05	0.00760389	Traf2/B2m/Rsad2/H2-K1/H2-D1/H2-Q6
Antigen processing and presentation of peptide antigen via MHC class Ib	GO:0002428	5.3637E-05	0.00825673	B2m/H2-K1/H2-D1/H2-Q6
Positive regulation of lymphocyte mediated immunity	GO:0002708	6.9783E-05	0.00926763	Traf2/B2m/H60c/Shld2/Rsad2/H2-K1/H2-D1/H2-Q6
T cell mediated immunity	GO:0002456	7.5255E-05	0.00926763	Traf2/B2m/Spn/H60c/Rsad2/H2-K1/H2-D1/H2-Q6
Positive regulation of adaptive immune response based on somatic recombination of immune receptors built from immunoglobulin superfamily domains	GO:0002824	7.5255E-05	0.00926763	Traf2/B2m/Shld2/Rsad2/H2-K1/H2-D1/H2-Q6/Cd274
Viral genome replication	GO:0019079	7.5255E-05	0.00926763	Oasl2/Ifitm3/Isg15/Oas1c/Oas1b/Bst2/Rsad2/Eif2ak2

Supplementary Table 1. Gene ontology analysis of RNA sequencing data from the non-lesional skin of OTULIN$^{E\text{-}KO}$ and skin of WT mice. Functional annotation by gene ontology of significantly up-regulated genes in OTULIN$^{E\text{-}KO}$ non-lesional skin tissue compared to floxed control mice (WT) skin tissue. For over-representation (ORA) tests, the enrichGO function was used with standard parameters defined by cut-off on p-value and logFoldChange from gene expression data from five 3-week old mice. 20 out of 61 enriched gene sets shown.

Biological process	GO ID	P-value	P-value (adj.)	Gene ID
Defense response to other organism	GO:0098542	1.33916E-23	5.3727E-20	Serpinb3a/S100a8/S100a9/2610528A11Rik/Ddx60/Rtp4/Irf7/Ifit1/Bst2/Oasl2/Dhx58/Parp14/Il1b/Tmem173/Il1f6/Shmt2/Ifih1/Ifitm3/Krt16/Eif2ak2/Oas2/Stat2/Cxcl9/Mmp12/H60c/Trex1/Il33/Bcl3/Dtx3l/Gbp2/Stat1/Ddx58/Cd14/Rsad2/Gbp7/Il1f9/Lcn2/Il4ra/Krt6a/Herc6/Ccl3/Il1f8/Slfn8/Parp9/Adar/Il1f5/Tlr3/Gch1/H2K1/Tap1/Trim30a/Defb1/Cxcl10/Cxcl16/Lgals9/Zc3h12a/Stx11/Slfn9/Nmi/Ccl8/Zbp1/Trim25/Clec4a2/Adam8/Samhd1/Krt1/Rnf19b/Nectin2/C1qa/Arg1/Trim27/Klk7/C3/Socs1/Gbp3/Cgas/Txk/Iigp1
Innate immune response	GO:0045087	2.87125E-21	5.7597E-18	Serpinb3a/S100a8/S100a9/Irf7/Ifit1/Bst2/Oasl2/Dhx58/Parp14/Tmem173/Il1f6/Shmt2/Ifih1/Ifitm3/Krt16/Eif2ak2/Oas2/Stat2/Mmp12/H60c/Trex1/Dtx3l/Gbp2/Stat1/Ddx58/Cd14/Rsad2/Gbp7/Il1f9/Lcn2/Herc6/Ccl3/Il1f8/Parp9/Adar/Il1f5/Tlr3/Gch1/Tap1/Trim30a/Defb1/Cxcl16/Lgals9/Stx11/Nmi/Ccl8/Zbp1/Trim25/Clec4a2/Adam8/Samhd1/Krt1/Rnf19b/Nectin2/C1qa/Arg1/Trim27/C3/Socs1/Gbp3/Cgas/Txk/Iigp1
Response to virus	GO:0009615	2.25552E-16	3.0164E-13	Ddx60/Rtp4/Irf7/Ifit1/Bst2/Oasl2/Dhx58/Il1b/Tmem173/Ifi27l2a/Ifih1/Ifitm3/Eif2ak2/Oas2/Stat2/Cxcl9/Mmp12/Trex1/Il33/Bcl3/Dtx3l/Stat1/Ddx58/Rsad2/Lcn2/Slfn8/Parp9/Adar/Tlr3/Trim30a/Cxcl10/Lgals9/Zc3h12a/Slfn9/Zbp1/Trim25/Samhd1/Cgas
Peptide cross-linking	GO:0018149	7.53991E-16	7.5625E-13	Sprr2d/Sprr1b/Sprr2f/Lce3c/Sprr2h/Sprr2e/Lce1g/Lce3b/Lce3e/Sprr2i/Sprr2g/Lce1k/Sprr2a3/Lce3a/Lce1e/Lce1f/Lce3f/Krt1/Lce1j/Tgm1/Lce3d
Defense response to virus	GO:0051607	1.33035E-15	1.0675E-12	Ddx60/Rtp4/Irf7/Ifit1/Bst2/Oasl2/Dhx58/Il1b/Tmem173/Ifih1/Ifitm3/Eif2ak2/Oas2/Stat2/Cxcl9/Mmp12/Trex1/Il33/Dtx3l/Stat1/Ddx58/Rsad2/Slfn8/Parp9/Adar/Tlr3/Trim30a/Cxcl10/Zc3h12a/Slfn9/Zbp1/Trim25/Samhd1/Cgas
Response to bacterium	GO:0009617	1.91904E-14	1.2832E-11	2610528A11Rik/Ifi44/Ifit1/Usp18/Dhx58/Il1b/Il1f6/Eif2ak2/Oas2/AA467197/Cxcl9/Slfn2/Bcl3/Gbp2/Stat1/Cd14/Cd274/Lrat/Cxcl2/Gbp7/Il1f9/Lcn2/Cd52/Krt6a/Herc6/Il1f8/Slfn4/Litaf/Il1f5/Ly6a/Gch1/Ppard/H2-K1/B2m/Trim30a/Defb1/Cxcl10/Cxcl16/Lgals9/Zc3h12a/Ptgs2/Psmb9/Ncl/Il1rn/Junb/Cmpk2/Rnase1/Klk7/C3/Gbp3/Iigp1
Immune effector process	GO:0002252	7.52624E-14	4.3136E-11	Serpinb3a/Ddx60/Rtp4/Irf7/Ifit1/Bst2/Oasl2/Dhx58/Il1b/Tmem173/Ifih1/Ifitm3/Eif2ak2/Oas2/Stat2/Cxcl9/Mmp12/H60c/Trex1/Il33/Bcl3/Dtx3l/Stat1/Ddx58/Rsad2/Il4ra/Slfn8/Parp9/Adar/Tlr3/H2-K1/Tap1/B2m/Trim30a/Cxcl10/Lgals9/Exo1/Zc3h12a/Stx11/Slfn9/H2D1/Zbp1/Trim25/Samhd1/Krt1/Rnf19b/Nectin2/Myb/Il7r/Clcf1/C1qa/Foxp3/Arg1/Traf2/Dnase1l3/Klk7/H2-T22/C3/Cgas
Keratinocyte differentiation	GO:0030216	1.68639E-13	8.4573E-11	Krt6b/Sprr2d/Sprr1b/Krt16/Sprr2f/Lce3c/Sprr2h/Sprr2e/Lce1g/Lce3b/Lce3e/Sprr2i/Sprr2g/Lce1k/Krt6a/Sprr2a3/Lce3a/Lce1e/Krt17/Lce1f/Ptgs2/Casp14/Lce3f/Lce1j/Sfn/Tgm1/Cnfn/Lce3d
Keratinization	GO:0031424	1.04115E-12	4.6412E-10	Krt6b/Sprr2d/Sprr1b/Krt16/Sprr2f/Sprr2h/Sprr2e/Sprr2i/Krt6a/Krt17/Casp14/Sfn/Tgm1/Cnfn
Response to interferon-beta	GO:0035456	1.16914E-11	4.6906E-09	Ifit1/Bst2/Tmem173/Ifitm3/Ifi202b/Trex1/Gbp2/Stat1/Igtp/Xaf1/Ifi47/Cdc34/Gbp3/Iigp1
Skin development	GO:0043588	2.11257E-11	7.7051E-09	Krt6b/Sprr2d/Sprr1b/Krt16/Sprr2f/Lce3c/Sprr2h/Sprr2e/Lce1g/Lce3b/Lce3e/Sprr2i/Sprr2g/Lce1k/Krt6a/Sprr2a3/Flg/Lce3a/Lce1e/Ppard/Krt17/Lce1f/Ptgs2/Gorab/Gba/Casp14/Dbi/Lce3f/Krt1/Lce1j/Sfn/Grhl3/Tgm1/Inhba/Cnfn/Lce3d/Ctsl
Regulation of defense response	GO:0031347	5.32927E-11	1.7818E-08	Serpinb3a/S100a8/S100a9/Ddx60/Irf7/Usp18/Dhx58/Parp14/Il1b/Tmem173/Mmp12/H60c/Trex1/Il33/Dtx3l/Stat1/Ddx58/Ldlr/Ccl3/Parp9/Adar/Tlr3/Ppard/Tap1/Lgals9/Psmb4/Zc3h12a/Nmi/Zbp1/Ptgs2/Adam8/Samhd1/Ctss/Krt1/Ccr5/Nectin2/Clcf1/Foxp3/Arg1/Dnase1l3/Klk7/C3/Socs1/D1Ertd622e/Cgas/Txk
Regulation of cytokine production	GO:0001817	6.55944E-11	2.0243E-08	Ddx60/Irf7/Dhx58/Aif1/Il1b/Tmem173/Il1f6/Ifih1/Eif2ak2/Mmp12/Trex1/Il33/Bcl3/Stat1/Ddx58/Cd14/Cd274/Rsad2/Il1f9/Il4ra/Ccl3/Il1f8/Litaf/Il1f5/Tlr3/B2m/Trim30a/Lgals9/Zc3h12a/Nmi/Ptgs2/Atf4/Hilpda/Gba/Clec4a2/Hif1a/Adam8/Casp8/Tspo/Capn2/Ccr5/Ncl/Il1rn/Foxp3/Hsp90ab1/Arg1/Traf2/Trim27/C3/Socs1/D1Ertd622e/Cgas/Txk
Epidermal cell differentiation	GO:0009913	1.17495E-10	3.3671E-08	Krt6b/Sprr2d/Sprr1b/Krt16/Sprr2f/Lce3c/Sprr2h/Sprr2e/Lce1g/Lce3b/Lce3e/Sprr2i/Sprr2g/Lce1k/Krt6a/Sprr2a3/Lce3a/Lce1e/Krt17/Lce1f/Ptgs2/Casp14/Lce3f/Lce1j/Sfn/Sult2b1/Tgm1/Cnfn/Lce3d
Epidermis development	GO:0008544	1.28768E-10	3.4441E-08	Krt6b/Sprr2d/Sprr1b/Krt16/Sprr2f/Lce3c/Sprr2h/Sprr2e/Lce1g/Lce3b/Lce3e/Sprr2i/Sprr2g/Lce1k/Krt6a/Sprr2a3/Flg/Lce3a/Lce1e/Ppard/Krt17/Lce1f/Ptgs2/Gorab/Casp14/Dbi/Lce3f/Lce1j/Sfn/Sult2b1/Grhl3/Tgm1/Zfp750/Inhba/Cnfn/Lce3d/Ctsl
Positive regulation of cytokine production	GO:0001819	4.46392E-10	1.1193E-07	Ddx60/Irf7/Dhx58/Aif1/Il1b/Tmem173/Il1f6/Ifih1/Eif2ak2/Mmp12/Il33/Bcl3/Stat1/Ddx58/Cd14/Cd274/Rsad2/Il1f9/Il4ra/Ccl3/Il1f8/Il1f5/Tlr3/B2m/Lgals9/Ptgs2/Atf4/Hilpda/Hif1a/Adam8/Casp8/Ccr5/Ncl/Il1rn/Foxp3/Traf2/C3/Cgas/Txk
Cellular response to interferon-beta	GO:0035458	1.6312E-09	3.8043E-07	Ifit1/Tmem173/Ifi202b/Trex1/Gbp2/Stat1/Igtp/Ifi47/Cdc34/Gbp3/Iigp1
Regulation of immune response	GO:0050776	1.70679E-09	3.8043E-07	Serpinb3a/Irf7/Dhx58/Parp14/Il1b/Tmem173/Mmp12/H60c/Trex1/Il33/Ddx58/Cd274/Rsad2/Il4ra/Parp9/Adar/Tlr3/H2K1/Tap1/B2m/Lgals9/Psmb4/Zc3h12a/H2D1/Nmi/Zbp1/Adam8/Samhd1/Krt1/Nectin2/Myb/Il7r/Clcf1/C1qa/Samsn1/Foxp3/Eif2b3/Arg1/Traf2/Trim27/Klk7/H2-T22/C3/Socs1/Cgas/Txk/Vav3
Regulation of response to biotic stimulus	GO:0002831	2.53674E-09	5.3565E-07	Serpinb3a/Ddx60/Irf7/Dhx58/Parp14/Il1b/Tmem173/Mmp12/H60c/Trex1/Dtx3l/Stat1/Ddx58/Cd274/Parp9/Adar/Tap1/Lgals9/Zc3h12a/Nmi/Zbp1/Adam8/Samhd1/Nectin2/Arg1/Klk7/Socs1/Cgas/Txk
Cytokine-mediated signaling pathway	GO:0019221	2.7938E-09	5.6044E-07	Irf7/Parp14/Il1b/Il1f6/Ifitm3/Oas2/Stat2/Cxcl9/Mmp12/Trex1/Stat1/Cxcl2/Il1f9/Cd300lf/Ccl3/Il1f8/Parp9/Adar/Il1f5/Il2rb/Cxcl10/Nmi/Ccl8/Zbp1/Samhd1/Il1rn/Arg1/Txndc17/Traf2/Cib1/Socs1/Txk/Iigp1

Supplementary Table 2. Gene ontology analysis of RNA sequencing data from the lesional skin of OTULIN$^{E\text{-}KO}$ and skin of WT mice. Functional annotation by gene ontology of significantly up-regulated genes in OTULIN$^{E\text{-}KO}$ lesional skin tissue compared to floxed control mice (WT) skin tissue. For over-representation (ORA) tests, the enrichGO function was used with standard parameters defined by cut-off on p-value and logFoldChange from gene expression data from five 3-week old mice. 20 out of 249 enriched gene sets shown.
