# Supplementary material for: OTULIN inhibits RIPK1-mediated keratinocyte necroptosis to prevent skin inflammation in mice
Source: Nat Commun. 2021 Oct 8;12:5912. doi: 10.1038/s41467-021-25945-1 (PMC8501112; doi:10.1038/s41467-021-25945-1)
Supplement: Supplementary file 3 — Reporting Summary [file 41467_2021_25945_MOESM3_ESM.pdf]

nature research

Corresponding author(s): Manolis Pasparakis

Reporting Summary

Nature Research wishes to improve the reproducibility of the work that we publish. This form provides structure for consistency and transparency in reporting. For further information on Nature Research policies, see Authors & Referees and the Editorial Policy Checklist.

Statistical parameters

When statistical analyses are reported, confirm that the following items are present in the relevant location (e.g. figure legend, table legend, main text, or Methods section).

n/a	Confirmed	
☐	☒	The exact sample size (*n*) for each experimental group/condition, given as a discrete number and unit of measurement
☐	☒	An indication of whether measurements were taken from distinct samples or whether the same sample was measured repeatedly
☐	☒	The statistical test(s) used AND whether they are one- or two-sided<br>*Only common tests should be described solely by name; describe more complex techniques in the Methods section.*
☒	☐	A description of all covariates tested
☐	☒	A description of any assumptions or corrections, such as tests of normality and adjustment for multiple comparisons
☐	☒	A full description of the statistics including central tendency (e.g. means) or other basic estimates (e.g. regression coefficient) AND variation (e.g. standard deviation) or associated estimates of uncertainty (e.g. confidence intervals)
☐	☒	For null hypothesis testing, the test statistic (e.g. *F*, *t*, *r*) with confidence intervals, effect sizes, degrees of freedom and *P* value noted<br>*Give P values as exact values whenever suitable.*
☒	☐	For Bayesian analysis, information on the choice of priors and Markov chain Monte Carlo settings
☒	☐	For hierarchical and complex designs, identification of the appropriate level for tests and full reporting of outcomes
☒	☐	Estimates of effect sizes (e.g. Cohen's *d*, Pearson's *r*), indicating how they were calculated
☐	☒	Clearly defined error bars<br>*State explicitly what error bars represent (e.g. SD, SE, CI)*

Our web collection on statistics for biologists may be useful.

Software and code

Policy information about availability of computer code

Data collection	No software was used for data collection.
Data analysis	Statistical analysis was performed with GraphPad Prism V6. Quantification of dead cell numbers was performed using GraphPad Prism V6. For analysis of the RNAseq data, Illumina adapters were clipped off the raw reads using cutadapt with standard parameters and a minimum read length of 35 after trimming (shorter reads were discarded). QuantSeq specific features were subsequently removed from the trimmed reads following the workflow described in https://rstudio-pubs-static.s3.amazonaws.com/171024_bd9c5ea013a4465cbceb9d435033eadd.html. Trimmed and cleaned reads were mapped to a concatenation of the mouse genome (Mus_musculus.GRCm38.dna.chromosome.*.fa.gz, downloaded from ftp://ftp.ensembl.org/pub/release-100/fasta/mus_musculus/dna/) and the ERCC92 Spike In sequences (downloaded from https://assets.thermofisher.com/TFS-Assets/LSG/manuals/ERCC92.zip), using subread-align version v2.0.1 with parameters -t 0 -d 50 -D 600 --multiMapping -B 5 . For counting, only high-quality uniquely mapping matches were retained, using samtools view -hb -q 30 -F 256. These matches were then combined into a count table using featureCounts with parameters -F "GTF" -t "exon" -g "gene_id" --minOverlap 20 -M --primary -O -J -T 4. Differential Gene Expression Analysis was done in R-4.0.0, using package DESeq2 (https://bioconductor.org/packages/release/bioc/html/DESeq2.html). Before heatmap visualization, gene counts were converted to Counts Per Million (CPM) and the CPM values were scaled by log10 (adding a pseudocount of 0.1). Heatmaps were drawn using the R package pheatmap (https://www.rdocumentation.org/packages/pheatmap/versions/1.0.12/topics/pheatmap), with parameters cluster_cols=FALSE and show_rownames=FALSE.<br>The individual heatmap shows genes that were significant in DESeq2 at a given p-value cutoff and had a logFoldChange of 1 or larger. Differential Gene Expression analysis and visualization was carried out in R-4.0.0, using package clusterProfiler (http://yulab-smu.top/clusterProfiler-book/). Individual DESeq2 results were tested one at a time. For over-representation (ORA) tests, which compare a pre-

defined subset of genes to the universe of all genes, the enrichGO function was used with standard parameters. The query gene subset for ORA analysis was defined by cutoffs on p-value and logFoldChange.

For manuscripts utilizing custom algorithms or software that are central to the research but not yet described in published literature, software must be made available to editors/reviewers upon request. We strongly encourage code deposition in a community repository (e.g. GitHub). See the Nature Research guidelines for submitting code & software for further information.

Data

Policy information about availability of data

All manuscripts must include a data availability statement. This statement should provide the following information, where applicable:
- Accession codes, unique identifiers, or web links for publicly available datasets
- A list of figures that have associated raw data
- A description of any restrictions on data availability

The RNA sequencing data discussed in this publication have been deposited in NCBI's Gene Expression Omnibus and are accessible through GEO Series accession number GSE (GSE180024) [https://www.ncbi.nlm.nih.gov/geo/query/acc.cgi?acc=GSE180024]. The source data underlying the graphs shown in the Figures are provided as a Source Data file. Uncropped images and replicates of immunoblots presented in the figures are included in the Source Data file.

Field-specific reporting

Please select the best fit for your research. If you are not sure, read the appropriate sections before making your selection.

[x] Life sciences [ ] Behavioural & social sciences [ ] Ecological, evolutionary & environmental sciences

For a reference copy of the document with all sections, see nature.com/authors/policies/ReportingSummary-flat.pdf

Life sciences study design

All studies must disclose on these points even when the disclosure is negative.

Sample size	Based on our previous experience on skin disease mouse models (doi: 10.26508/lsa.202000956) we aimed to analyze a sufficient number of animals per group (minimum 5 animals) to evaluate differences between different groups of genetically modified mice. In vitro experiments with isolated primary cells were performed at least 2-3 times from cell batches from individual mice (biological replicates) to confirm reproducibility.
Data exclusions	No data was excluded from the analysis
Replication	For our in vivo studies we analyzed a sufficient number of animals (5-25 mice per group) to ensure that the results obtained are consistent. For in vitro studies in primary cells we replicated all experiments in at least 3 independently isolated primary cell batches from individual mice (biological replicates). In each experiment at least 3 replicates were analysed (technical replicates).
Randomization	No specific method of randomization had been used to select animals. We compared groups of mice with different genotypes to assess the effect of specific genetic mutations in the phenotype. Group allocation was thus determined by the genotype of the mice. We did not specifically control for covariates in this study.
Blinding	Quantification of the pathology by macroscopical scoring of the back and tail skin was performed blindly.

Reporting for specific materials, systems and methods

Materials & experimental systems

n/a	Involved in the study
[x]	[ ] Unique biological materials
[ ]	[x] Antibodies
[x]	[ ] Eukaryotic cell lines
[x]	[ ] Palaeontology
[ ]	[x] Animals and other organisms
[x]	[ ] Human research participants

Methods

n/a	Involved in the study
[x]	[ ] ChIP-seq
[x]	[ ] Flow cytometry
[x]	[ ] MRI-based neuroimaging

Antibodies

Antibodies used

-monoclonal rabbit anti-p-IκBα, clone 5A5, Cat. No. 2859, Cell Signaling Technology; dilution 1:1000 for WB, Lot. No. 17
-polyclonal rabbit anti-IκBα, Cat. No. sc-371, Santa Cruz Biotechnology; dilution 1:1000 for WB, Lot. No. K1315
-monoclonal rabbit anti-p-p65, Cat. No. 3033, Cell Signaling Technology; dilution 1:1000 for WB, Lot. No. 16
-polyclonal rabbit anti-p65, Cat . No sc-372, Santa Cruz Biotechnology; dilution 1:1000 for WB, Lot. No. K0415
-polyclonal rabbit anti-p-SAPK/JNK (T183/T185), Cat. No. 4668, Cell Signaling Technology; dilution 1:1000 for WB, Lot. No. 15
-polyclonal rabbit anti-SAPK/JNK, Cat. No. 9252, Cell Signaling Technology; dilution 1:1000 for WB, Lot. No. 17
-polyclonal rabbit anti-p-p38, Cat. No. 9211, Cell Signaling Technology; dilution 1:1000 for WB, Lot. No. 25
-polyclonal rabbit anti-p38, Cat. No. 9212, Cell Signaling Technology; dilution 1:1000 for WB, Lot. No. 17
-polyclonal rabbit anti-p-p44/42 MAPK (ERK1/2), Cat. No. 9191, Cell Signaling Technology; dilution 1:1000 for WB, Lot. No. 29
-polyclonal rabbit anti-p44/42 MAPK (ERK1/2), Cat. No. 9102, Cell Signaling Technology; dilution 1:1000 for WB, Lot. No. 27
-polyclonal rabbit anti-OTULIN, Cat no. 14127, Cell Signaling Technology; dilution 1:1000 for WB, Lot. No. 1
-polyclonal rabbit anti-SHARPIN, Cat no. 14626-1-AP; Proteintech; dilution 1:1000 for WB, Lot. No. 1
-human IgG anti M1-Ubiquitin, Genentech; dilution 1:1000 for WB, Clone 1F11/3F5/Y102L
-monoclonal mouse anti-Tubulin, Cat. No. T6074, Sigma-Aldrich; dilution 1:1000 for WB, Lot No. 118M4779

-sheep anti-mouse IgG horseradish peroxidase (HRP)-linked antibody, Cat. No. NA931, GE Healthcare, dilution 1:10000 for
-sheep anti-rabbit IgG conjugated to HRP antibody, Cat. No. NA934V, GE Healthcare, dilution 1: 10000 for WB
-donkey anti-human IgG (H+L) conjugated to HRP, Cat. No. 709-036-149, Jackson ImmunoResearch, dilution 1:5000 for WB

-polyclonal rabbit anti-cleaved Caspase3, Cat. No. 9661, Cell Signaling Technology; dilution for IHC 1:1000, Lot. No. 433
-monoclonal rat anti-cleved Caspase 8, Cat. No. ALX-804-447, Alexis; dilution 1:600 for IHC, Lot No. 08271911
-monoclonal rat anti-Ki67, Cat. No. M724901, DAKO, dilution for IHC 1:1000, Lot No. TEC-3

-polyclonal rabbit anti-Keratin 10, Cat. No. PRB-159P, Covance, dilution 1:300 for IHC
-polyclonal rabbit anti-Keratin 14, Cat. No. MS-115, Neomarkers, dilution1:400 for IHC
-polyclonal rabbit anti-Keratin 6, Cat. No. PRB-169P, Covance, dilution 1:1000 for IHC
-monoclonal rat anti-F4/80, Cat. No. MCA497, clone A3-1, AbD Serotec, dilution 1:1000 for IHC
-monoclonal rat anti-Gr-1, Cat. No. MCA2387GA, clone RB6-8C5, AbD Serotec, dilution 1:500 for IHC

-anti mouse Alexa 488- fluorescence-conjugated secondary Ab, Cat. No. A1101, Molecular Probes, dilution 1:800 for IHC
-anti rabbit Alexa 549- fluorescence-conjugated secondary Ab, Cat. No. A11012, Molecular Probes, dilution 1:800 for IHC
-anti rat Alexa 488- fluorescence-conjugated secondary Ab, Cat. No. A21210, Molecular Probes, dilution 1:800 for IHC

Validation

All primary antibodies used in this study were purchased from commercial sources and are fully validated by the respective Vendors. Validation data can be found on the respective Vendor websites for all antibodies.

Animals and other organisms

Policy information about studies involving animals; ARRIVE guidelines recommended for reporting animal research

Laboratory animals

The following mouse lines were used: Otulinfl/fl (32), K14-Cre (25), Tnfr1fl/fl (33), Ripk1D138N/D138N (34), Ripk3-/- (35), Mlkl-/- (24), Faddfl/fl (36), Ripk3-/- (35) and MyD88fl/fl (37). All these mouse lines have been described before in the respective publications cited. The experiments were performed on mice backcrossed into the C57BL/6 genetic background for at least five generations. In all experiments, littermates carrying the loxP-flanked alleles but not expressing Cre recombinase were used as wild-type controls. Mice were analysed between 3 and 50 weeks of age. Mice used in this study were maintained in the animal facility of the CECAD Research Center, University of Cologne, in individually ventilated cages (Greenline GM500; Tecniplast) at 22°C (± 2°C) and a relative humidity of 55% (± 5%) under 12-hour light cycle on sterilized bedding (Aspen wood, Abedd, Germany) and with access to sterilized commercial pelleted diet (Ssniff Spezialdiäten GmbH) and acidified water ad libitum. The microbiological status was examined as recommended by Federation of European Laboratory Animal Science Associations (FELASA) and the mice were free of all listed pathogens. All animal procedures were conducted in accordance with European, national, and institutional guidelines, and protocols were approved by local government authorities (Landesamt für Natur, Umwelt und Verbraucherschutz Nordrhein-Westfalen). Animals requiring medical attention were provided with appropriate care and were sacrificed when reaching pre-determined criteria of disease severity.

Wild animals

The study did not involve wild animals

Field-collected samples

The study did not involve samples collected from the field.
